# In the sea slug *Melibe leonina* the posterior nerves communicate stomach distention to inhibit feeding and modify oral hood movements

**DOI:** 10.3389/fphys.2022.1047106

**Published:** 2022-11-23

**Authors:** Colin Anthony Lee, Winsor Hays Watson

**Affiliations:** ^1^ Department of Biological Sciences, University of New Hampshire, Durham, NH, United States; ^2^ Neuroscience Program, University of Illinois at Urbana-Champaign, Urbana, IL, United States

**Keywords:** gastropod, nudibranch, invertebrate, motivation, arousal, satiation, feeding, behavior

## Abstract

The sea slug *Melibe leonina* is an excellent model system for the study of the neural basis of satiation, and previous studies have demonstrated that stomach distention attenuates feeding. Here we expanded on this work by examining the pathway communicating stomach distention to the central nervous system and the effects of distention on motor output. We found that the posterior nerves (PN), which extend posteriorly from the buccal ganglia and innervate the stomach, communicate stomach distention in Melibe. PN lesions led to increased feeding duration and food consumption, and PN activity increased in response to stomach distention. Additionally, the percentage of incomplete feeding movements increased with satiation, and PN stimulation had a similar impact in the nerves that innervate the oral hood. These incomplete movements may be functionally similar to the egestive, food rejecting motions seen in other gastropods and enable *Melibe* to remain responsive to food, yet adjust their behavior as they become satiated. Such flexibility would not be possible if the entire feeding network were inhibited.

## Introduction

In recent years the sea slug *Melibe leonina* has emerged as a promising species in which to study the neuronal regulation of behavioral state. It feeds using rhythmic movements of its oral hood to capture both planktonic prey (Watson and Trimarchi, 1992) and organisms on the kelp and sea grass on which it tends to reside (Watson et al., 2021), and it can feed while either stationary or crawling. Both its rhythmic feeding movements (Watson and Trimarchi, 1992) and locomotor activity (Newcomb et al., 2014) can be easily quantified, and the expression of these movements changes with time of day (Newcomb et al., 2014) or hunger state (Lee and Watson, 2016). Like all heterobranch gastropods, *Melibe* has a simple nervous system with a small number of large, individually identifiable neurons. However, unlike most heterobranchs *Melibe* lacks a buccal mass and complicated chewing mechanics, leading to an exceptionally simple (∼30 neurons) buccal ganglion (Trimarchi and Watson, 1992). Finally, it is an organism about which we have transcriptomic (Cook et al., 2018) and peptidomic (Lee et al., 2021) information, and whose daily rhythms have been thoroughly documented (Newcomb et al., 2014).

**FIGURE 1:**
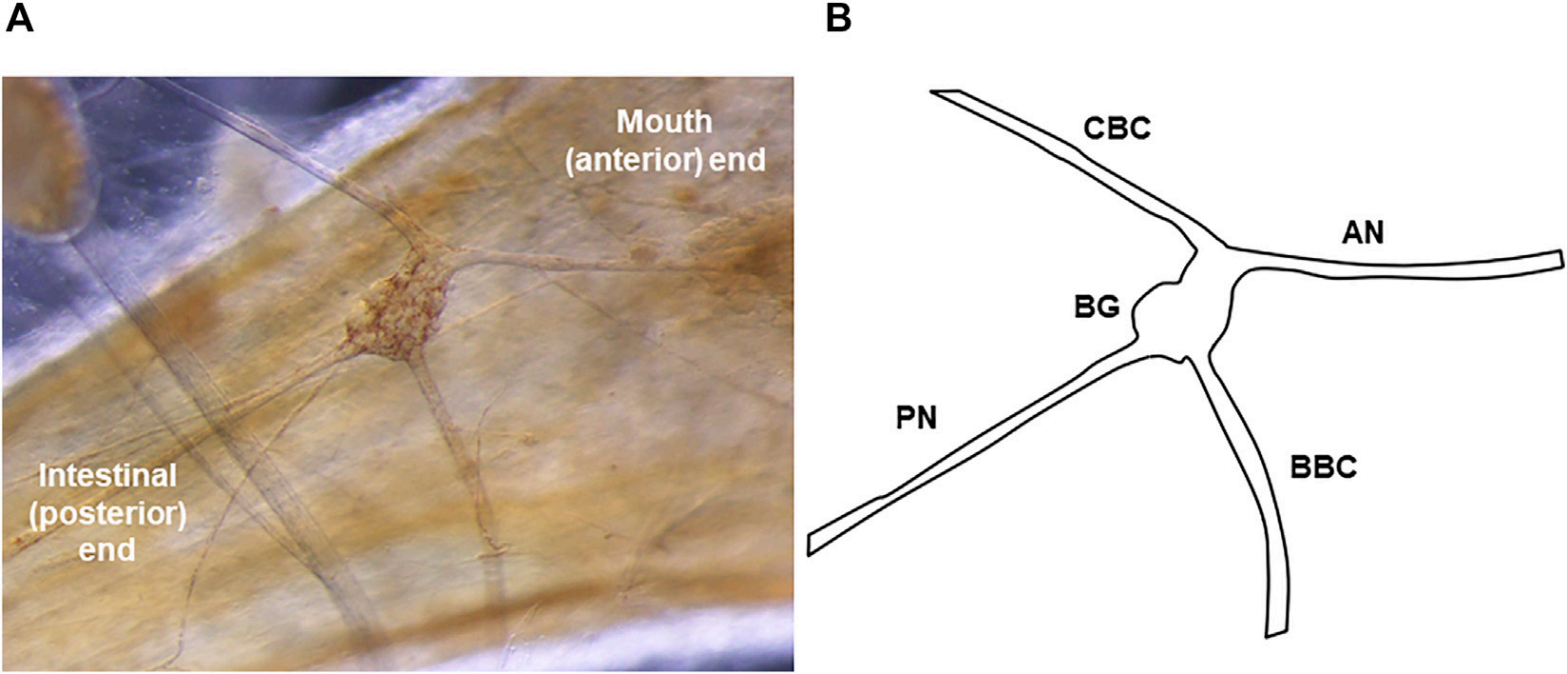
The *Melibe* buccal ganglion. **(A)** Picture of the buccal ganglion on the esophagus. A portion of the pedal ganglion, which is part of the fused cerebral-pleural-pedal complex, is visible in the top left. **(B)** Diagram of the ganglion and its nerves. The PN leaves the BG posteriorly and runs towards the stomach and intestines. In neurophysiological experiments examining the effect of stomach distention on activity, recordings were made from the severed distal end of the PN (conveying afferent signals from the stomach).

Both time of day and hunger state influence behavioral arousal in *Melibe*, but our understanding of the neuronal mechanisms by which hunger does so remains limited. As a nocturnal animal, *Melibe* shows increased arousal during the night, with increased feeding and locomotion (Newcomb et al., 2014), and greater responsiveness to excitatory neurotransmitters (Watson et al., 2020). Its expression of key clock genes also peaks during this active period (Duback et al., 2018), consistent with what is seen across the metazoa (von Schantz and Archer, 2003; Nitabach and Taghert, 2008). A major regulator of hunger state in *Melibe* is stomach distention: as the volume in the stomach increases individuals become less likely to feed and move, until they are eventually satiated, and cease feeding altogether (Lee and Watson, 2016). This pattern has also been observed in other gastropods (e.g. *Pleurobranchaea californica* (Gillette et al., 2000), *Lymnaea stagnalis* (Dyakonova et al., 2015), and *Aplysia californica* (Kupfermann, 1974)) and other invertebrates (e.g. the fly *Phormia meigen* (Green, 1964) and the leech *Hirudo medicinalis* (Lent and Dickinson, 1987)). Stomach distention is conveyed by nerves that innervate the esophagus and stomach (Dethier and Gelperin, 1967; Kupfermann, 1974; Susswein and Kupfermann, 1975; Croll et al., 1987), and in several gastropods reductions in the motivation to feed are accompanied by reconfiguration of feeding networks to produce egestive, food rejecting motions (London and Gillette, 1986; Jing et al., 2007; Crossley et al., 2018). In *Melibe*, the most likely pathway for this signaling is *via* the posterior nerves (PN) of the buccal ganglia, which emanate from the buccal ganglia and innervate the esophagus and stomach (Figure 1 (Trimarchi and Watson, 1992)). The primary goal of this study was to test this hypothesis.

In this study we demonstrated that PN activity changes in response to stomach distention, and that PN signaling is necessary for normal satiation of feeding behavior. Additionally, we found that PN signaling attenuates feeding not merely by reducing the output from the feeding network, but by shortening feeding movements, so they do not cause consumption of food. These data demonstrate that the PNs are important regulators of the motivation to feed in *Melibe*.

## Methods

### Animals

Adult *Melibe leonina* (21.3 ± 2.69 g) were acquired from eelgrass beds near the University of Washington’s Friday Harbor Laboratories (FHL) in the Puget Sound, WA and in Monterey Bay, California, and either maintained at FHL or shipped to the University of New Hampshire, Durham, NH. The subjects used at FHL were housed in sea tables with flow through seawater, while those used at the University of New Hampshire were maintained in an aquarium with recirculating seawater, at approximately 13°C, until needed for experiments.

### Feeding assays

To determine the effect of PN signaling on the motivation to feed, experiments were performed in which feeding rate over time was assessed for both *Melibe* with PN lesions (described in the next section) and control animals. Individual *Melibe* were placed in circular buckets (30 cm diameter) located within a larger tank of aerated seawater. The buckets had small mesh “windows” that allowed water exchange with the larger tank but prevented food from escaping. Tanks were located in a 13°C cold room that was kept on a 24-h light/dark cycle, with 10–14 h of light per day, depending on the season. After subjects acclimated to the buckets for 24 h, sufficient newly hatched *Artemia* spp. (brine shrimp) were added to the bucket to yield a density of approximately 3,000 *Artemia*/L. All trials began between 10 and 11 a.m. and *Melibe* were allowed to feed undisturbed for approximately 24 h. A black and white low light sensitive camera suspended directly above the buckets captured feeding activity, and recordings were obtained from approximately 1 hour before *Artemia* addition to 24 h post-addition. Camera outputs were digitized, time-stamped, and recorded on a Macintosh computer using the video capture software Gawker, which took one picture every second and streamed the images together at a rate of ten frames per second (Supplemental Video S1).

During the subsequent video analysis, the number of feeding motions performed per minute was counted for the entire experiment. *Melibe* feeds using rhythmic movements termed oral hood closures (OHCs), which consist of an oral hood closing phase (in which the hood comes forward and closes, capturing a bolus of water) and a tilt and squeeze swallowing phase (in which the closed hood is tilted back and the water is squeezed out through tentacles that are used to capture prey); see Watson and Trimarchi (Watson and Trimarchi, 1992) for a complete description of these phases. Subjects routinely produced incomplete feeding movements, performing only the oral hood closing phase, and likely not ingesting captured prey (Trimarchi and Watson, 1992). For comparisons between PN lesioned and control individuals these incomplete motions were ignored, and only the complete, ingestive OHCs were quantified. In a separate analysis of control individuals, both complete (Supplemental Video S2) and incomplete OHCs (Supplemental Video S3) were quantified.

### PN lesions

*Melibe* were pinned out dorsally on a Sylgard-coated dish, with a single pin through the foot and two through the oral hood, and viewed under a dissecting microscope. A single incision was made in the skin directly above the central nervous system (CNS), exposing the fused cerebral, pedal, and pleural ganglia, the buccal ganglia, and PNs. Both PNs were then either cut with scissors or torn with tweezers. Incisions were sewn up with sterile sutures, and the subjects were given at least 5 days to recover. After this recovery period lesioned animals were fed as described in the previous section, and their feeding activity was compared to that of control animals. Half of the controls received sham operations, in which incisions were made and sutured, but PNs were left intact. There was no difference in the feeding activity between sham-operated (n = 4) and unoperated controls (n = 8). The baseline feeding rate for controls was 0.28 ± 0.12 OHC/min and 0.66 ± 0.22 OHC/min for sham-operated *Melibe* (T (10) = 1.90, *p* = 0.087). The maximum feeding rate was 2.15 ± 0.59 OHC/min for unoperated animals vs. 2.75 ± 0.17 OHC/min for those that were sham-operated (T (10) = 0.76, *p* = 0.47).

### Quantification of food consumed during a satiating meal

Six lesioned and six control *Melibe* were fed to satiation with *Artemia*. After their feeding rate had returned to baseline, each individual’s stomach was removed, and the number of *Artemia* in it was estimated. For estimates, the contents of the stomach were emptied into a known volume of seawater and stirred thoroughly to produce a homogenous concentration of *Artemia*. Three separate 1 ml aliquots were drawn up, and the number of *Artemia* in each aliquot was counted separately by two different people. Numbers were averaged between the measurements and used to estimate the total number of *Artemia* in the larger sample.

### Neurophysiology

**FIGURE 2:**
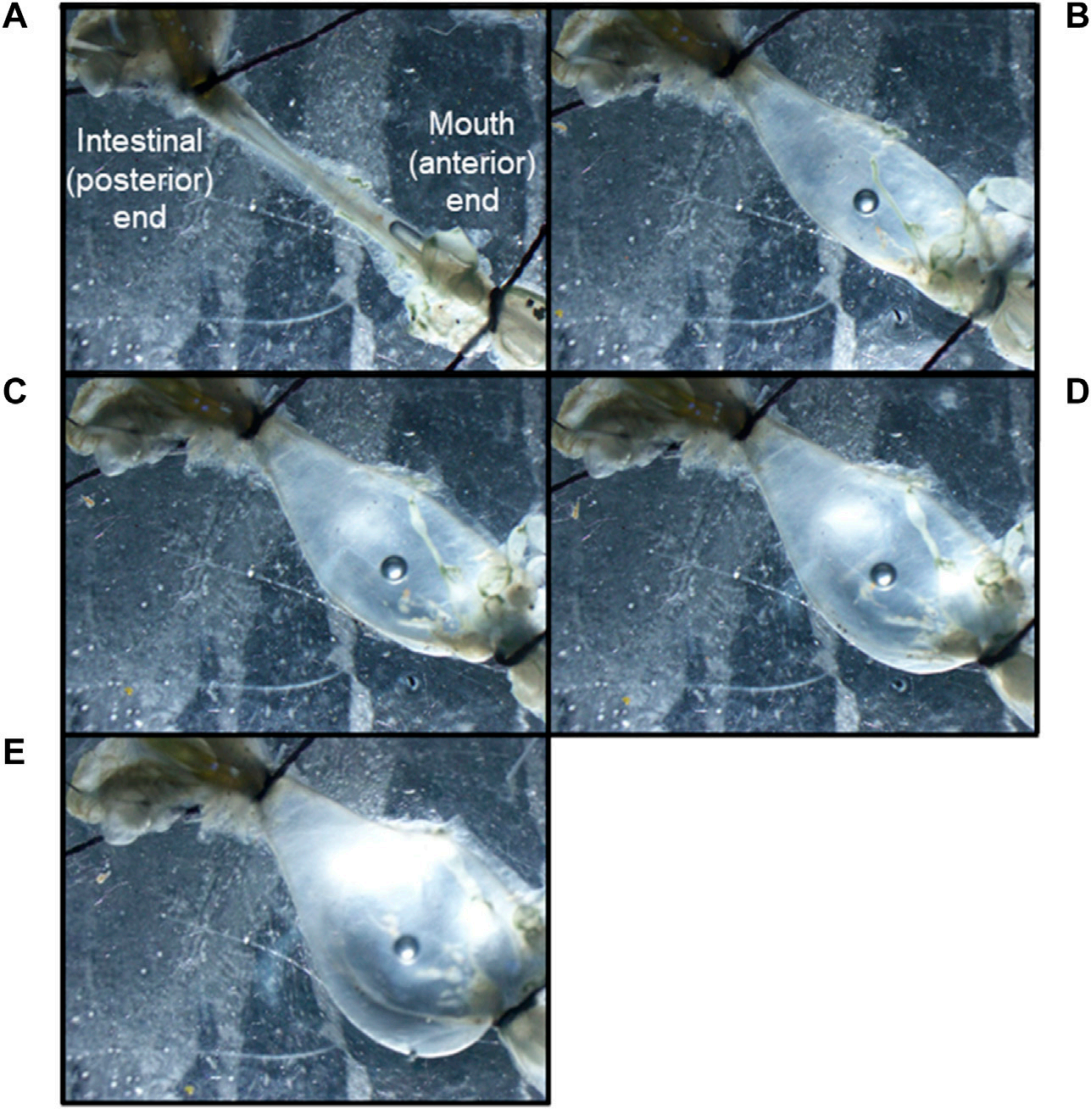
A *Melibe* mouth, esophagus and stomach preparation distended to the following degrees: **(A)** empty, **(B)** ¼ full, **(C)** ½ full, **(D)** ¾ full, **(E)** fully distended. Note the cannula held in place by a thread in the bottom right corner of image A, and the tied-off junctions between the stomach and intestines in all the images.

Extracellular neurophysiological recordings were carried out using both isolated CNS and semi-intact preparations. The semi-intact preparation was used to assess the effects of stomach distention on nervous system activity. For this preparation, the intact mouth, esophagus, stomach, intestine, and buccal ganglia were removed, pinned out in a Sylgard-coated dish, and continuously perfused with 13°C seawater. A cannula was inserted through the mouth and esophagus into the stomach, and a thread was used to tighten the junction of the mouth and esophagus to the cannula. Another thread was used to close the junction between the stomach and the intestine (Figure 2). Seawater was injected through the cannula to distend the stomach to one of four different diameters (1/4, 1/2, 3/4, maximum), and recordings were obtained from the PN (n = 6). To record the action potentials traveling *via* the PN from the stomach to the buccal ganglia, the nerve was severed near the connection to the buccal ganglion and recordings were obtained from the cut distal end.

To assess the effects of PN activity on feeding motor output, extracellular recordings were obtained from the oral hood nerves (OHN) of the cerebral ganglion during sustained PN stimulation. For these recordings, the CNS was cut almost entirely away from the body, with only a piece of the esophagus retained to ensure that the buccal ganglia were not lost. The esophageal portion was pinned in a Sylgard-lined dish, and suction electrodes were attached to the PN and one to three OHNs. Recordings were carried out for several hours, alternating between ∼30 min without PN stimulation and 20 min with PN stimulation. The PN was stimulated at 10 Hz 5 V. Fictive feeding can be identified in isolated *Melibe* brains by recording OHN bursts that have the same frequency as the oral hood movements of intact animals. Bursts identified using the following criteria: 1) at least 10 s in duration; 2) spiking at a rate of at least 5 Hz and at a rate that was at least twice that of the baseline spiking rate. For activity to be defined as a spike, it needed to be at least twice the voltage of the background noise.

Recordings were made with suction electrodes made of either pulled borosilicate capillaries or polyethylene tubing. Signals were amplified and filtered with an AM Systems Microelectrode AC Amplifier (AM-systems, Sequim, WA), digitized with an AD Instruments Powerlab 4/30 (AD Instruments, Dunedin, New Zealand), and displayed with Labchart software (AD Instruments, Dunedin, New Zealand). Stimuli were triggered using Labchart’s Stimulator function. Changes in firing rate over time were determined by counting number of spikes/min using Labchart software.

### Statistics

For the experiment comparing feeding rate over time in control and lesioned individuals, data were normalized by subtracting the baseline rate from the rate at a given time point. In all graphs, data are shown as the mean ± the standard error of means. All statistical tests were performed with the software Prism (Graphpad, Boston, MA). A two-way repeated measures ANOVA with Sidak post-test was used to compare feeding rate over time between control and lesioned individuals. One-way repeated measures ANOVAs with Tukey post-tests were used to assess changes in complete vs. incomplete OHCs over time and PN responses to stomach distention. Changes in OHN activity were assessed using paired t-tests, and differences in the number of *Artemia* consumed between lesioned and control *Melibe* were assessed with unpaired t-tests.

## Results

### Posterior nerve lesions led to increases in feeding behavior

**FIGURE 3:**
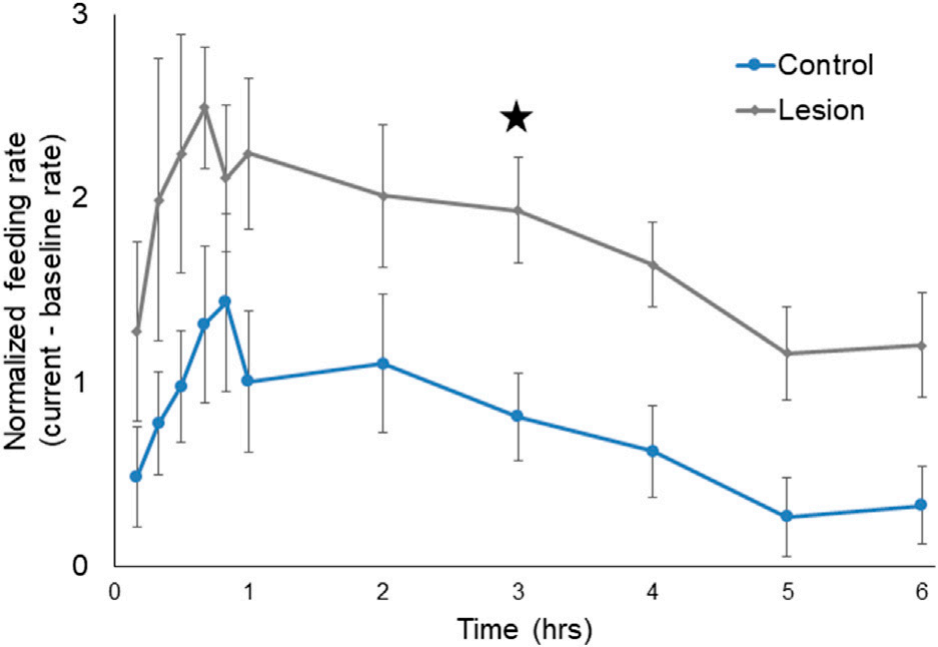
Normalized feeding rate over time for both control *Melibe* and individuals with PN lesions. For the graph rates were calculated in 10-min bins for the first hour, and for every hour thereafter; for statistical analysis rates were calculated for every hour. In the third hour the feeding rate was significantly elevated for lesioned individuals compared to controls (indicated by star).

PN lesioned *Melibe* (n = 6) fed more than controls (n = 12) (Figure 3). The two groups displayed statistically significant differences in normalized feeding rate by both time (F (5,80) = 6.96, *p* < 0.001) and treatment group (F (1,16) = 9.56, *p* = 0.0070), although the interaction between the two variables was not significantly different (F (5,80) = 0.13, *p* = 0.99, two-way repeated measures ANOVA). Lesioned *Melibe* fed at a significantly greater rate in the third hour of their feeding bout (1.94 ± 0.29 OHC/min vs. 0.82 ± 0.24 OHC/min; *p* = 0.042, Sidak’s Multiple Comparison Test). However, there was no significant difference in their maximum feeding rates (lesioned: 2.84 ± 0.30 OHC/min; controls: 1.94 ± 0.40 OHC/min; T (16) = 1.64, *p* = 0.12, unpaired *t*-test). Thus, PN lesions did not increase the initial or peak feeding intensity, but prolonged the duration of food arousal.

**FIGURE 4:**
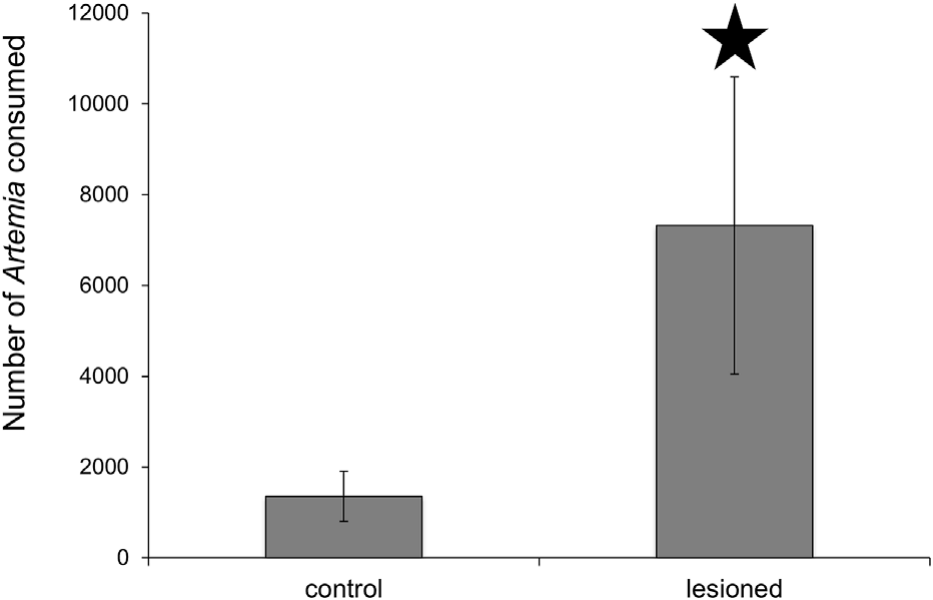
Assessment of stomach contents following feeding to satiation. *Melibe* with posterior nerve lesions consumed significantly more *Artemia* than controls. *Artemia* were less than 24 h old, and at this age are typically 500 μm in length (Wurstbaugh and Maciej Gliwicz, 2001).

To verify that the lesioned *Melibe* consumed more prey, the stomachs of six lesioned and six control *Melibe* were removed after they had ceased feeding (8.9 h for lesioned individuals, 4.1 h for controls), and the *Artemia* in the stomachs were counted (Figure 4). Lesioned *Melibe* consumed significantly more *Artemia* (7323 ± 3278 *Artemia* for lesioned and 1358 ± 590 *Artemia* for controls; *p* = 0.04, *t*-test).

### The frequency of complete, but not incomplete, OHCs decreased over time

**FIGURE 5:**
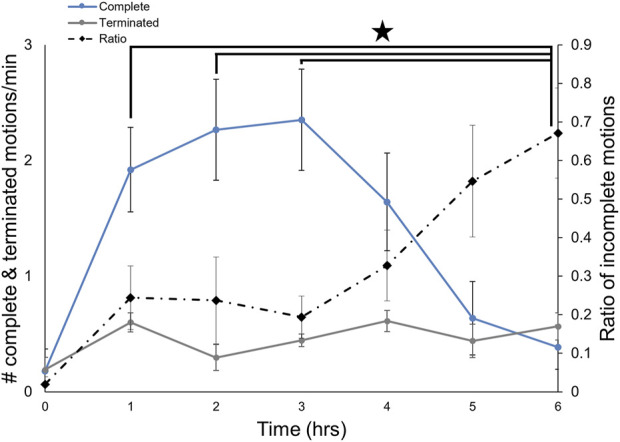
Changes in the types of OH movements during a feeding bout. The number of incomplete feeding motions remained constant over the course of a feeding bout, whereas the number of complete ones decreased after 3 hours. The frequency of incomplete movements was significantly greater at 6 h than for the first 3 hours.

Like many gastropods, *Melibe* can produce multiple feeding related movements, performing both complete OHCs, which lead to food ingestion, and incomplete ones, which likely do not lead to ingestion (Watson and Trimarchi, 1992; Wurstbaugh and Maciej Gliwicz, 2001; Crossley et al., 2018). To determine if the proportion of complete OHCs changes over the course of a feeding bout, we recorded incomplete vs. complete motions for a group of intact *Melibe* for 6 h (Figure 5, n = 8). While the rate of incomplete motions changed minimally over the course of the feeding period, the rate of complete motions dropped dramatically throughout the feeding bout, leading to a significantly increased ratio of incomplete motions for the final 2 hours of feeding compared to the first 3 h (F (6,42) = 5.81, *p* < 0.001, *p* < 0.05 for each comparison; one-way repeated measures ANOVA with Tukey post-test).

### Stomach distention increases PN activity

**FIGURE 6:**
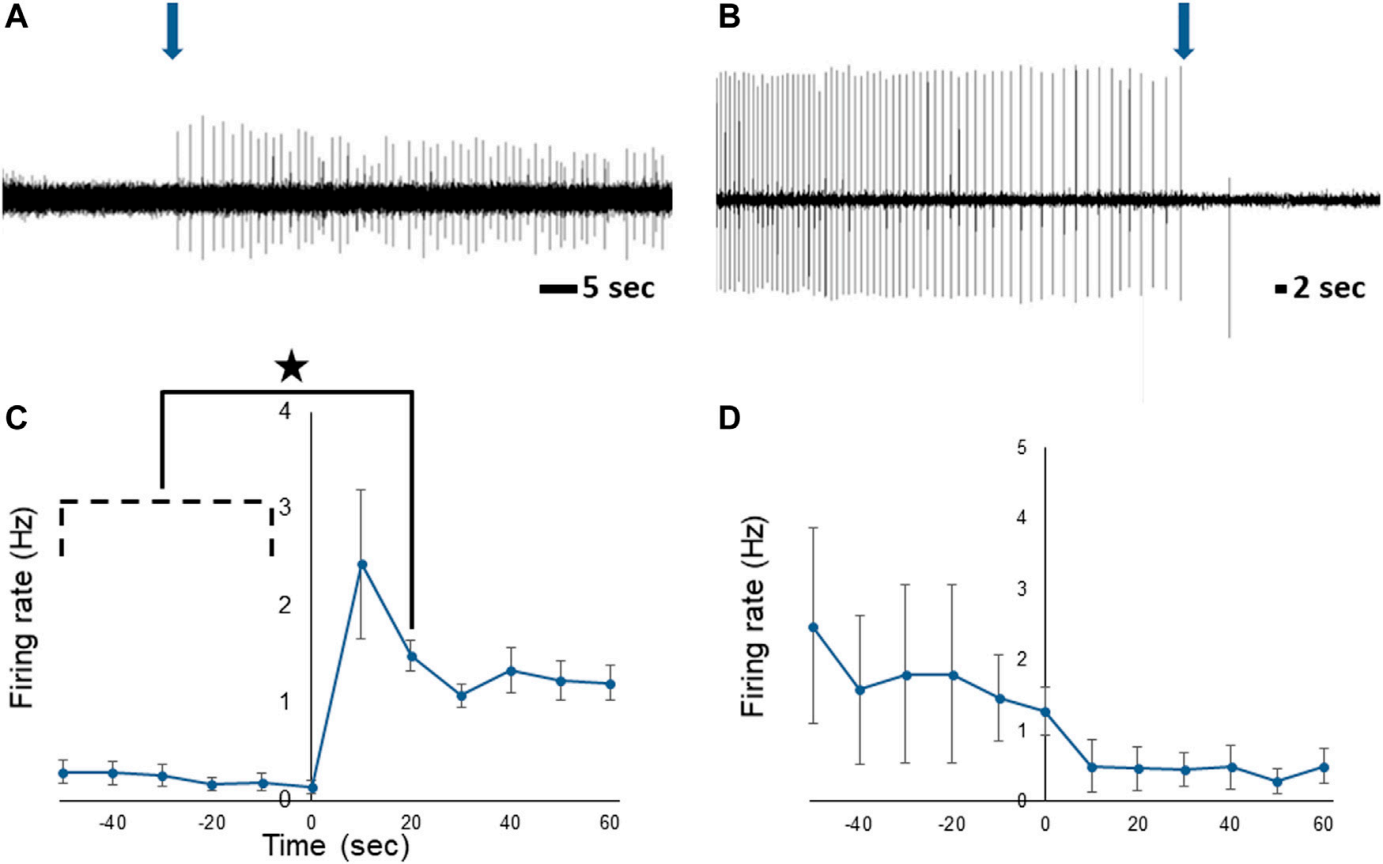
Response of the *Melibe* PN to stomach distention. **(A)** Representative neurophysiological recording showing the increase in spiking starting at the onset of distention (arrow). **(B)** Representative decrease in activity when the stomach was deflated (arrow). **(C)** Average spike frequency during the onset of distension, firing was significantly elevated compared to baseline at 10–20 s. **(D)** Average spike frequency before and after deflation of the stomach.

To determine if the PNs communicate information about stomach fullness to the CNS, extracellular recordings were obtained from the PN while the stomach was artificially inflated with seawater. Although there was activity in the PNs even when the stomach was empty, spike frequency changed in response to distention (Figures 6A, C) and was significantly increased compared to baseline rates 10–20 s after the start of distension (n = 5; F (11,44) = 2.39, *p* < 0.021; one-way repeated measures ANOVA with Tukey post-test). After the stomach was deflated PN activity showed a trend toward a decrease in firing rate (Figures 6B, D), although the decrease was not statistically significant (n = 6; F (11,55) = 1.853, *p* = 0.067; one-way repeated measures ANOVA).

**FIGURE 7:**
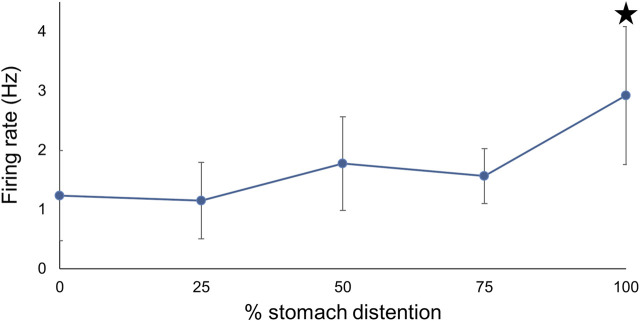
The relationship between PN firing rate and different levels of stomach distention. Maximal distention significantly increased firing compared to baseline, ¼, and ¾ distention.

To assess the effects of different levels of prolonged stomach distention on PN nerve activity we obtained PN recordings while the stomach was inflated to ¼, ½, ¾, and 100% of its maximum for at least 30 min at each distention level (Figure 7). During sustained distention multiple units burst rhythmically, and the overall firing rate increased (n = 6; F (4,25) = 5.98, *p* = 0.0025, one-way repeated measures ANOVA with Tukey post-test). Complete distention of the stomach caused a significant increase in posterior nerve firing compared to baseline, ½, and ¾ distention (*p* < 0.05 for each comparison) suggesting that partial stomach distention has a minimal impact on feeding behavior in *Melibe*.

### PN signaling alters feeding-related activity in the CNS

**FIGURE 8:**
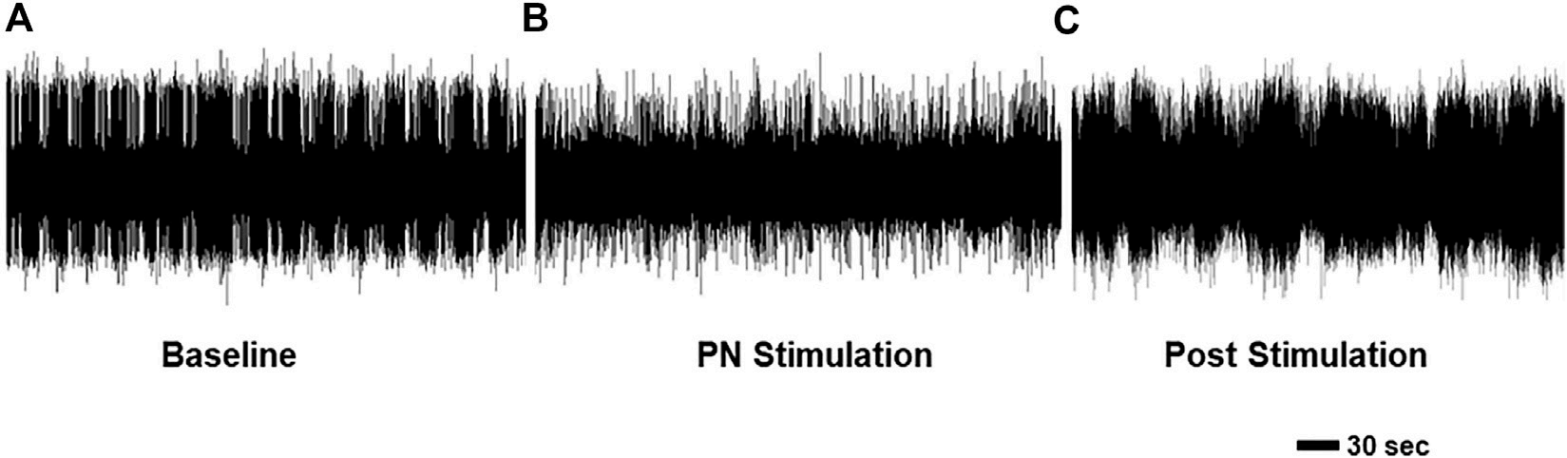
Recordings from an OHN showing activity **(A)** before, **(B)** during, and **(C)** after PN stimulation. Note the PN stimulation led to distinct bursting, which likely represents a change in the movements of the oral hood.

Finally, we examined the effect of PN stimulation on patterns of OHN activity. These nerves emerge from the cerebral ganglion, cause contraction of the oral hood, the first phase of feeding behavior (Watson and Trimarchi, 1992), and produce spontaneous rhythmic bursts in isolated perparations (Figure 8A. 1.19 ± 0.21 OHCs/min, 21.70 ± 2.51 s duration). Long term recordings were performed during which we alternated between approximately 30 min without stimulation and 20 min with PN stimulation, and we found that stimulation significantly changed both the frequency (n = 4; F (2,6) = 60.24, *p* < 0.001, one-way repeated measures ANOVA with Tukey post-test) and duration of bursts F (2,6) = 9.74, *p* = 0.013, one-way repeated measures ANOVA with Tukey post-test). Specifically, stimulation of the PNs (Figure 8B) significantly reduced both the rate (0.56 ± 0.21 OHCs/min, 47.1% of baseline; *p* < 0.001) and duration (17.82 ± 2.12 s duration 82.1% of baseline; *p* = 0.049) of these bursts, with returns to baseline after stimulation ceased (Figure 8C; 1.08 ± 0.24 bursts/min, 90.8% of baseline; *p* = 0.98; 23.58 ± 2.44 s duration, 108.6% of baseline; *p* = 0.49). The shorter duration bursts that occurred during PN stimulation likely correspond to incomplete OHCs.

## Discussion

This study demonstrates a clear role for the PNs in the regulation of *Melibe* feeding behavior. Both behavioral and neurophysiological data indicate that stomach distention, a key factor in *Melibe* satiation (Lee and Watson, 2016), is communicated to the CNS by the PNs. The data also indicate that this signaling leads to three changes in feeding related activity: 1) a decrease in the overall number of feeding movements; 2) an increase in the ratio of incomplete, non-ingestive movements to normal complete movements; 3) a decrease in the rhythmic movements of the esophagus associated with swallowing. This suggests that satiation in *Melibe* is more complex than simple inhibition of the feeding network.

### The PNs facilitate the inhibition of feeding by stomach distention

Loss of PN signaling, after they were lesioned, dramatically prolonged the duration of feeding-related arousal in *Melibe*. Lesions led to increases in both the duration of feeding bouts and the amount of food consumed. These results were very similar to those previously obtained when Melibe stomachs were lesioned, so food could not distend the stomach (Lee and Watson, 2016). The initial intensity of feeding did not increase in PN lesioned individuals, suggesting that they were not hungrier or experiencing greater food-induced arousal at the start of a meal. Rather, they simply took longer to satiate. This change did not appear to be due to behavioral deficits caused by the lesions, as individuals were fully healed from the surgery when experiments were performed, were still capable of consuming food, and consumed more than sham operated individuals. Additionally, in lesioned individuals, we observed defecation and transport of gut contents to the diverticuli that run throughout the body, indicating that the difference in final stomach volume was not a product of changes in the processing of stomach contents. Similar results have been obtained during studies involving the posterior stomatogastric nerve in *Pleurobranchaea* (Croll et al., 1987) and the esophageal nerve in *Aplysia* (Jing et al., 2007), suggesting a common function for the nerves that innervate the gut in gastropods.

Activity in the PNs signals the CNS that the stomach is filling with prey, and the information transmitted is proportional to the stimulus. Increases in stomach fullness cause immediate increases in PN activity, and deflation of the stomach causes an immediate cessation of activity. Additionally, the firing rate from the PNs during sustained (>30 min) distention roughly correlates with the degree of distention. However, a significant increase in the PN firing rate does not take place until the stomach is 100% distended, which suggests that *Melibe* will continue to feed until the stomach fills to this degree.

### Satiation in Melibe is more complex than simple inhibition of feeding circuitry

Although satiation led to overall reductions in food-induced arousal, the feeding network still responded to food, but the response changed. Freely behaving *Melibe* continued to perform OHCs throughout a feeding bout, but the number of complete, ingestive ones decreased as their stomachs filled, whereas the incomplete, ineffective ones, remained unchanged. Similarly, the isolated CNS continued to produce rhythmic bursts during PN stimulation, but the duration of these bursts decreased. Therefore, as they start to become satiated, their feeding efforts become less effective and, while they do not start to egest prey, they also do not consume prey.

Other gastropods show a similar pattern as they satiate, producing egestive movements (Cropper et al., 2004; Wang et al., 2019), which are used to reject food in multiple contexts (e.g. (McManus et al., 2019)). It is unclear if these are homologous to *Melibe*’s incomplete OHCS, but they display functional similarities, as both are food-evoked movements of the feeding apparatus that do not result in consumption. *Aplysia* (Jing et al., 2007) and *Lymnaea* (Crossley et al., 2018) switch from ingestion to egestion as they satiate, and satiated *Pleurobranchaea* perform aversive behaviors in response to appetitive stimuli (Gillette et al., 2000). Mechanistic similarities also exist, as in *Aplysia* the shift to egestion is driven by the esophageal nerve, which, like the *Melibe* PN, innervates the gut and esophagus (Chiel et al., 1986). Studies in these species have also identified individual neurons involved in this shift. In both *Aplysia* (Jing et al., 2007) and *Lymnaea* (Crossley et al., 2018) an egestion specific interneuron becomes active, and in satiated *Aplysia* the multifunctional CBI2 interneuron, which can elicit both ingestive and egestive behaviors (Jing and Weiss, 2001), drives egestion. In *Pleurobranchaea* food avoidance conditioning biases individuals towards egestion, and this conditioning is accompanied by increases in the excitability of retraction phase neurons (London and Gillette, 1986), which in normal feeding behavior inhibit the protraction phase interneurons (London and Gillette, 1984). Similarly, in the land snail *Limax marginatus* feeding network oscillations increase following food avoidance conditioning (Kimura et al., 1998; Inoue et al., 2006). Thus, as gastropods satiate, they respond to palatable food in the same way they respond to unpalatable or toxic stimuli.

Why does satiation not merely manifest as simple inhibition of the feeding network, with no response of any type to food? Such inhibition can occur with defensive behaviors. In *Lymnaea* (Alania et al., 2004) and the nudibranch *Clione limacina* (Alania et al., 1999) the PlB neuron, which fires during defensive withdrawal, globally shuts down the feeding network, inhibiting neurons that act throughout the network, including the retraction phase ones that drive egestion. The answer likely lies in the complex and variable nature of the motivation to feed. Hunger state is not binary, but rather lies on a continuum (Susswein et al., 1976). On the hungry end of the continuum individuals feed robustly and primarily produce ingestive motions, whereas on the satiated end feeding responses are reduced and most movements are egestive or incomplete. Shifts away from ingestion allow individuals to flexibly move along this continuum. As they satiate, the number of non-ingestive movements decreases, but they can temporarily increase their feeding rate if food abundance or quality increases, in the same way that hungry individuals can temporarily perform egestive or incomplete feeding movements in response to an undesired food item.

This may be particularly important for *Melibe*, which filter feeds on patchy sources of planktonic prey, and which needs to be able to adaptively modify its feeding behavior in response to changes in food availability (Watson and Trimarchi, 1992). For example, a given bolus of seawater may hold toxic or unpalatable prey, so temporary expression of incomplete feeding motions allows individuals to clear undesirable material without ending a feeding bout. With inhibition as seen from the PlB neurons, feeding is inflexibly inhibited, and the feeding network cannot respond to changes in environmental condition. The existence of these separate mechanisms for feeding inhibition strongly indicates the utility of preserving some responsiveness within the feeding network, even with satiation.

### How do stomach distention and circadian clocks interact to regulate the motivation to feed?

In *Melibe*, which are nocturnal animals, the onset of darkness initiates locomotion and bouts of feeding (Newcomb et al., 2014). This conflicts with satiation, which inhibits feeding behavior and has been shown in other species to inhibit overall activity. Studies in *Melibe* and other gastropods suggest that serotonin (5-HT) is involved in these processes. In gastropods it acts *via* the metacerebral cells and other 5-HT-ergic cerebral neurons to strongly potentiate feeding (Weiss et al., 1975; Weiss and Kupfermann, 1976; Gillette and Davis, 1977; Granzow and Kater, 1977; McCrohan and Benjamin, 1980; Delaney and Gelperin, 1990; Kobatake et al., 1992), and in *Pleurobranchaea* the 5-HT content of the metacerebral cells is reduced following satiation (Hatcher et al., 2008). Additionally, synaptically connected 5-HT-ergic neurons are embedded in each of the major locomotor networks (Gillette, 2006; Dyakonova et al., 2015), and act to regulate overall behavioral arousal; inhibition of 5-HT neurons in one circuit correlates with inhibition in another (Dyakonova et al., 2015). 5-HT mediates the effects of light in animals across the Metazoa (Morin, 1999; Itoh and Igarashi, 2000; Saifullah and Tomioka, 2002), including *Aplysia* (Koumenis and Eskin, 1992) and the marine snail *Bulla gouldiana* (Whitmore and Block, 1996), and is generally more abundant during an animal’s active period (Itoh and Igarashi, 2000). In *Melibe*, 5-HT-ergic processes project to the buccal ganglion *via* the cerebral-buccal connective (Newcomb et al., 2006).

**FIGURE 9:**
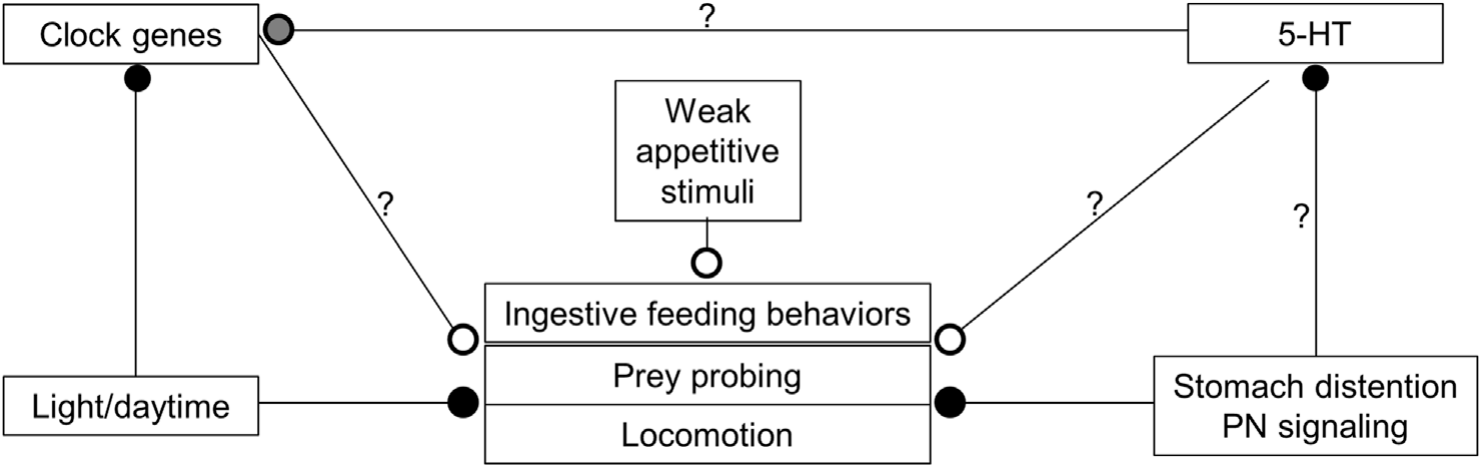
Model showing how various inputs might modulate *Melibe* feeding, locomotion, and overall arousal. Open circles are excitatory, closed circles are inhibitory. During the day, which is their quiescent period, 5-HT levels are reduced and light both inhibits active behaviors and reduces the expression of several clock genes. With the onset of darkness, clock genes are upregulated and 5-HT levels rise, leading to increased feeding, locomotion, and overall arousal. 5-HT may also have effects on clock gene expression (gray circle), perhaps serving to phase advance the circadian clock. When food is ingested it causes the stomach to distend, which is communicated to the NS by the PNs that innervate the stomach. This input reduces the motivation to feed, 5-HT levels, and locomotion. In the sea slug *Melibe leonina* the posterior nerves communicate stomach distention to inhibit feeding and modify oral hood movements.

Overall, hunger state, time of day, and the abundance of food act together to modulate the expression of feeding (Figure 9). During the day, 5-HT levels are likely lower, which combines with input from the circadian clock and inhibition from light, to reduce activity and responsiveness to the presence of prey in the water column. When night falls, 5-HT levels rise, circadian clock input changes, and it is dark, which leads to increased activity, and responsiveness to the same food-related stimuli that are present in the day. However, when individuals consume a satiating meal, even though they might still be in an active state, feeding is diminished.

## Conclusion and future directions

This study demonstrates that stomach distension reduces the expression of rhythmic feeding behavior in *Melibe* and the state of distension is communicated to the NS *via* the posterior nerve. Satiating signals likely act alongside circadian clock cues to regulate overall behavioral arousal, and it is likely that 5-HT plays an important role. Further studies might eventually identify the neural circuits for feeding and swallowing and thus make it possible to determine how PN activity modifies their activity so there is a tendency for less feeding and more incomplete, likely egestive, movements. Additionally, although clear similarities in feeding behavior can be seen across gastropods, there is diversity in prey choice, feeding apparatus morphology, feeding mechanics, and gut morphology. Future work may also examine if these traits lead to interspecific variation in mechanisms of satiation.

## Data Availability

The raw data supporting the conclusions of this article will be made available by the authors, without undue reservation.

## Abbreviations

5-HT, Serotonin; CNS, Central Nervous System; OHC, Oral Hood Closure; OHN, Oral Hood Nerve; PN, Posterior Nerve.
