# Supplementary material for: In the sea slug Melibe leonina the posterior nerves communicate stomach distention to inhibit feeding and modify oral hood movements
Source: Front Physiol. 2022 Nov 23;13:1047106. doi: 10.3389/fphys.2022.1047106 (PMC9727288; doi:10.3389/fphys.2022.1047106)
Supplement: Supplementary file 2 [file DataSheet1.PDF]

Supplementary Material

Supplemental video 1: Sample time lapse video showing four *Melibe* feeding within individual arenas.

Supplemental video 2: Example of complete OHCs. Two OHCs are performed in the video.

Supplemental video 3: Example of incomplete OHCs. Two such motions are performed.
